# Co-Expression of Molecular Chaperones to Enhance Soluble aPD-L1 scFv Production in *Escherichia coli* BL21(DE3)

**DOI:** 10.3390/life16101664

**Published:** 2026-10-03

**Authors:** Sarinthip Khamthiang, Chonlaphon Juntama, Aussara Panya, Pachara Sattayawat

**Affiliations:** 1 Department of Biology, Faculty of Science, Chiang Mai University, Chiang Mai 50200, Thailand; sarinthip_k@cmu.ac.th (S.K.); chonlaphon_j@cmu.ac.th (C.J.); aussara.pan@cmu.ac.th (A.P.); 2 Cell Engineering for Cancer Therapy Research Group, Chiang Mai University, Chiang Mai 50200, Thailand

**Keywords:** cancer immunotherapy, antibody, microbial cell factory, Programmed Death-Ligand 1

## Abstract

Production of commercial antibodies commonly relies on mammalian expression platforms; however, these are associated with high production costs and technical challenges during large-scale cultivation. In this study, we aimed to develop an *Escherichia coli*-based platform for the production of an anti-PD-L1 single-chain variable fragment, hereafter aPD-L1 scFv. Expression of aPD-L1 scFv in *E. coli* BL21(DE3) resulted in extensive inclusion body formation regardless of the expression temperatures at 16 °C, 30 °C, and 37 °C, with approximately 84–93% accumulating in the insoluble fraction. To improve its solubility, co-expression with several molecular chaperones, namely DnaK-DnaJ-GrpE and GroES-GroEL, was investigated. Optimization of L-arabinose concentrations as an inducer for DnaK-DnaJ-GrpE expression demonstrated that induction at 0.15 mg/mL increased aPD-L1 scFv solubility from 5.13% to 23.97%, although the difference was not statistically significant. Moreover, His-tag purification yielded 14.78 µg/mL and 18.62 µg/mL without and with molecular chaperone co-expression, respectively. The binding activity of purified aPD-L1 scFv was confirmed by ELISA, which showed comparable binding signals following chaperone-assisted expression. This indicated that the functional activity was retained. Altogether, these findings suggest that molecular chaperone co-expression has the potential to increase the soluble production of functional aPD-L1 scFv in *E. coli*, supporting the feasibility of bacterial expression systems as a cost-effective platform for recombinant antibody fragment production.

## 1. Introduction

Microbial cell factories for the production of recombinant proteins, particularly those based on *Escherichia coli*, offer several advantages, including rapid growth rates, ease of genetic manipulation, cost-effective cultivation, and suitability for large-scale production. However, challenges are generally associated with two main factors. First, *E. coli* lacks the machinery required for post-translational modifications, which can result in the synthesis of inactive proteins, particularly those of eukaryotic origins [1]. Second, rapid protein expression, which is considered an advantage of using the *E. coli* platform, can also lead to protein misfolding and aggregation [2]. An inclusion body refers to a highly aggregated form of protein that is typically formed in the cytoplasm; when a secretion vector is used, inclusion bodies may also form in the periplasmic space or extracellular environment of *E. coli* [3]. The formation of inclusion bodies often results in non-functional proteins that require solubilization and subsequent refolding to restore their native conformation and biological activity before downstream applications [4]. Antibody production using *E. coli* as an expression host commonly employs single-chain variable fragments (scFvs) or heavy-chain variable domains (VHHs), as these antibody formats do not require complex post-translational modifications such as glycosylation [5]. However, a major challenge regarding the formation of inclusion bodies remains, as it can result in improperly folded and non-functional antibody fragments.

Several factors can influence inclusion body formation, which are mostly related to the high rate of protein synthesis. For example, the use of elevated expression temperatures, high inducer concentrations, and strong promoter systems frequently leads to rapid protein synthesis [2]. Several reports attempted to alleviate inclusion body formation using engineering strategies such as (i) the use of fusion tags and signal peptides [6,7] and (ii) the use of molecular chaperones [8]. While fusion tags and signal peptides require modification of the target protein sequences, co-expression of chaperones from a separate plasmid represents a more versatile and broadly applicable approach. Molecular chaperones are proteins that play many crucial roles in the cell. For example, the Seventeen kilodalton protein (Skp) is a well-characterized periplasmic molecular chaperone that binds to unfolded or partially folded outer membrane proteins [9] and prevents aggregation. Other well-reported chaperones are DnaK-DnaJ-GrpE (Hsp70) and GroES-GroEL (Hsp60) systems, which play important roles in facilitating protein folding [10]. Previously, the co-expression of molecular chaperones with scFvs in *E. coli* has been reported, including the use of Skp [11] and different combinations of DnaK-DnaJ-GrpE and GroES-GroEL [12].

Anti-PD-L1 (hereafter aPD-L1) is an antibody that binds to Programmed Death-Ligand 1 (PD-L1) on cancer cells. This binding prevents the interaction between PD-L1 and its receptor PD-1 on T cells, thereby restoring T cell-mediated immune responses [13]. Recently, this antibody has gained attention for its use in immunotherapy for several cancers [14]. Currently, three aPD-L1 monoclonal antibodies, atezolizumab, durvalumab, and avelumab, have been approved for the treatment of various cancers, including non-small cell lung cancer, urothelial carcinoma, and Merkel cell carcinoma, by blocking the PD-1/PD-L1 immune checkpoint and restoring antitumor T-cell responses [15,16,17]. Mammalian expression systems are used to produce these antibodies, which come with complicated production procedures and relatively high manufacturing costs. Recombinant antibody fragments, especially single-chain variable fragments (scFvs), have emerged as attractive alternatives as they maintain the antigen-binding specificity of full-length antibodies, have a smaller molecular size, better tissue penetration, and can be produced at an economical level in microbial hosts such as *E. coli* [18]. Therefore, we aimed to develop *E. coli* as a production platform for aPD-L1. To achieve this, co-expression of aPD-L1 scFv with molecular chaperones is proposed to enhance the soluble expression of aPD-L1 scFv in *E. coli*. In this study, the DnaK-DnaJ-GrpE and GroES-GroEL systems were selected based on their well-established roles in protein folding, as well as their representation as complete molecular chaperone systems. Furthermore, optimization of expression conditions, purification of the recombinant aPD-L1 scFv, and functional validation of the produced antibody fragment were systematically investigated.

## 2. Materials and Methods

### 2.1. E. coli Strains and Plasmid Construction

In this work, *E. coli* DH5α was used as a propagating strain for all plasmids and *E. coli* BL21(DE3) was used for the expression. A pET-22b(+) plasmid was used as an expression vector carrying the gene encoding the aPD-L1 scFv, hereafter pET-22b-aPD-L1, constructed via conventional restriction enzyme cloning. The gene sequence of aPD-L1 scFv was obtained from a previously reported aPD-L1 scFv [19]. A plasmid encoding molecular chaperones (DnaK-DnaJ-GrpE and GroES-GroEL), namely pG-KJE8, was purchased from Takara Bio (Kusatsu, Shiga, Japan). To assess the efficiency of molecular chaperones, *E. coli* BL21(DE3) co-transformed with pET-22b-aPD-L1 and pG-KJE8 was used for the co-expression of aPD-L1 scFv and molecular chaperones. As a control, *E. coli* BL21(DE3) harboring pET-22b-aPD-L1 and an empty pACYC vector (GoldBio, St. Louis, MI, USA) was used, which contains the same chloramphenicol resistance gene as pG-KJE8, to ensure consistent selection while expressing aPD-L1. All plasmids used in this study are listed in Table 1.

### 2.2. Protein Expression and Extraction

To evaluate the solubility and localization of aPD-L1 scFv, pET-22b-aPD-L1 was transformed into chemically competent *E. coli* BL21(DE3) cells by heat shock transformation. An overnight culture was inoculated into 25 mL of LB medium supplemented with 100 µg/mL ampicillin. The cells were cultivated at 37 °C, 180 rpm for 2 h (or until reaching an OD600 of 0.4–0.6), followed by induction with 0.5 mM isopropyl β-D-1-thiogalactopyranoside (IPTG) and incubation at 16 °C, 30 °C or 37 °C for 4 h. After induction, the culture was centrifuged at 3709× *g* for 5 min at room temperature to separate the cells from the culture supernatant. The resulting cell pellet was washed twice with 1× phosphate-buffered saline (PBS), resuspended in 10 mL 1× PBS, and lysed by sonication at 25% amplitude for 10 min (3 s on, 1 s off) on ice. The lysates were centrifuged at 4 °C, 10,304× *g* for 30 min. The resulting supernatant was collected as the soluble cell lysate, and the pellet was retained as the insoluble cell debris.

To assess the role of molecular chaperones, *E. coli* BL21(DE3) harboring pET-22b-aPD-L1 and pG-KJE8 was cultivated as described at the induction temperature of 30 °C; however, L-arabinose (0.15 and 4 mg/mL) and tetracycline (5 ng/mL) were added concurrently with 0.5 mM IPTG to induce the co-expression of molecular chaperones and aPD-L1 scFv.

### 2.3. Quantification and Qualification of Proteins

To quantify protein concentration, the Bradford assay (Bio-Rad, Hercules, CA, USA) was used following the manufacturer’s protocol. Total proteins were extracted and investigated via sodium dodecyl sulfate-polyacrylamide gel electrophoresis (SDS-PAGE) and Western blot analysis. Protein concentrations were adjusted to ensure equal amounts in each sample. Then, 20 µL of each sample was mixed with 5 µL of 5× reducing sample buffer, heated at 95 °C for 5 min, and loaded into each well. Proteins were separated by SDS-PAGE at 150 V for 90 min, with a prestained protein molecular weight marker (Bio-Helix, New Taipei City, Taiwan) included as a reference. After electrophoresis, gels were fixed and subsequently stained with Coomassie Brilliant Blue R-250 at room temperature with gentle agitation for 1 h. The gels were then destained overnight until the protein bands were clearly visualized. For Western blot analysis, proteins were transferred to a nitrocellulose membrane using a semi-dry transfer system at 85 mA for 45 min. The membrane was blocked with 5% (*w*/*v*) skim milk in 0.1% (*v*/*v*) TBST at room temperature with gentle agitation for 30 min to prevent non-specific binding. After blocking, the membrane was washed three times with 0.1% (*v*/*v*) TBST for 5 min each, then incubated at 4 °C for 16–18 h with anti-His primary antibody (1:1000, Thermo Fisher Scientific, Waltham, Massachusetts, USA; RRID:AB_557403). The membrane was washed and then incubated for 1 h at room temperature with HRP-conjugated goat anti-mouse IgG (1:1000, Jackson ImmunoResearch, West Eve, PA, USA; RRID:AB_2313567), followed by washing three times with 0.1% (*v/v*) TBST to remove unbound antibodies. Protein bands were visualized using a chemiluminescent substrate (Cytiva, Marlborough, MA, USA) and detected with the ImageQuant™ LAS 500 Imaging System. The intensity of the protein bands was quantified by densitometric analysis using ImageJ software (version 1.54g). Finally, the percentage of protein solubility was calculated as follows:

% Solubility = [Soluble protein/(Soluble protein + Insoluble protein)] × 100

### 2.4. Purification of His-Tagged aPD-L1 scFv

For purification purposes, the aPD-L1 scFv was fused with a 6× histidine-tag (His-tagged aPD-L1 scFv), allowing for subsequent purification using a His GraviTrap TALON His-tag affinity chromatography (Cytiva, Little Chalfont, Buckinghamshire, UK). The column was initially equilibrated with 10 mL of binding buffer (applied twice). A total of 8 mL of cell lysate was loaded onto the column and incubated for 10 min to allow binding before collecting the flow-through fraction. The average amount of total soluble protein loaded was approximately 1.5 mg, although slight variation occurred among biological replicates. To remove non-specifically bound proteins, the column was washed twice using 10 mL of binding buffer (Wash 1) followed by 10 mL of binding buffer supplemented with 5 mM imidazole (Wash 2), and each wash fraction was collected separately. Bound proteins were eluted stepwise using 1 mL of binding buffer containing increasing concentrations of imidazole (100 mM, 200 mM, and 400 mM), and each eluted fraction was collected separately. After elution, the column was washed with 10 mL of MES buffer followed by 10 mL of distilled water. An Amicon Ultra-4 10K (Merck Millipore, Carrigtwohill, County Cork, Ireland) centrifugal filter unit was used for protein buffer exchange into 1× PBS after purification. The protein concentration of the purified fractions was determined using the Bradford assay with bovine serum albumin (BSA) as the protein standard, and their purity was verified via SDS-PAGE and Western blot analysis, as previously mentioned. The purified proteins were stored at −20 °C.

### 2.5. Enzyme-Linked Immunosorbent Assay (ELISA)

An indirect ELISA was performed to evaluate the binding activity of purified aPD-L1 scFv against recombinant human PD-L1 protein. Briefly, to coat 96-well ELISA plates, 100 µL of recombinant human PD-L1 protein (Sino Biological, Beijing, China) (1 µg/mL in coating buffer) was added to each well and incubated at 4 °C for 16–18 h. The plates were washed three times with 100 µL of 1× PBS for 5 min each. The wells were blocked with 150 µL of 1% (*w*/*v*) BSA in PBS for 1 h at room temperature. After blocking, 100 µL of purified aPD-L1 scFv (20 µg/mL) was added, while positive control wells were treated with 100 µL of rabbit anti-PD-L1 monoclonal antibody (mAb) (0.625 µg/mL, ABclonal, China; RRID:AB_2862628), incubated at 37 °C for 1 h. Following incubation, plates were washed three times with 100 µL 0.1% (*v*/*v*) PBST (PBS containing 0.1% Tween-20), for 5 min each. Next, 100 µL of mouse anti-His IgG (1:1000 in PBS) was added to detect the purified aPD-L1 scFv wells, followed by incubation at 37 °C for 1 h. After a subsequent wash step, the corresponding secondary antibodies were added: 100 µL of HRP-conjugated goat anti-mouse IgG secondary antibody (1:1000 dilution in PBS; RRID:AB_2313567) for the purified aPD-L1 scFv wells, and HRP-conjugated goat anti-rabbit IgG (1:1000 dilution in PBS; RRID:AB_10015289) for the positive control wells. The plates were then incubated at 37 °C for 1 h and finally washed three times with 0.1% (*v*/*v*) PBST for 5 min each. For signal development, 50 µL of TMB substrate (Life Technologies, Frederick, MD, USA) solution was added to each well and incubated at room temperature for 3 min. The reaction was stopped by adding 50 µL of 0.5 M H_2_SO_4_. Absorbance was measured at 450 nm within 30 min using a microplate reader (EZ Read 2000, Biochrom, Cambridge, UK). Negative control wells, which contained 100 µL of 1× PBS instead of the purified aPD-L1 scFv, were used to evaluate the non-specific binding of detection antibodies. Similarly, blank wells were prepared by excluding the recombinant human PD-L1 protein during the coating step to determine the baseline background absorbance of the assay. Additionally, BSA (1 µg/mL) was used as an unrelated antigen control to assess non-specific binding of the purified aPD-L1 scFv.

### 2.6. Statistical Analysis

Data are presented as mean ± standard deviation (SD) from three biological replicates. Differences between two groups were analyzed using a two-sided Student’s *t*-test, whereas differences among three or more groups were analyzed using one-way analysis of variance (ANOVA) followed by Tukey’s multiple comparison test. Significant differences are indicated by asterisks (*p* ≤ 0.05, ** *p* ≤ 0.01, *** *p* ≤ 0.001 and **** *p* ≤ 0.0001), while ns indicates no significant difference. For multiple comparisons, different lowercase letters indicate significant differences among groups (*p* ≤ 0.05).

## 3. Results

### 3.1. Expression of aPD-L1 scFv in E. coli BL21(DE3)

**Figure 1:**
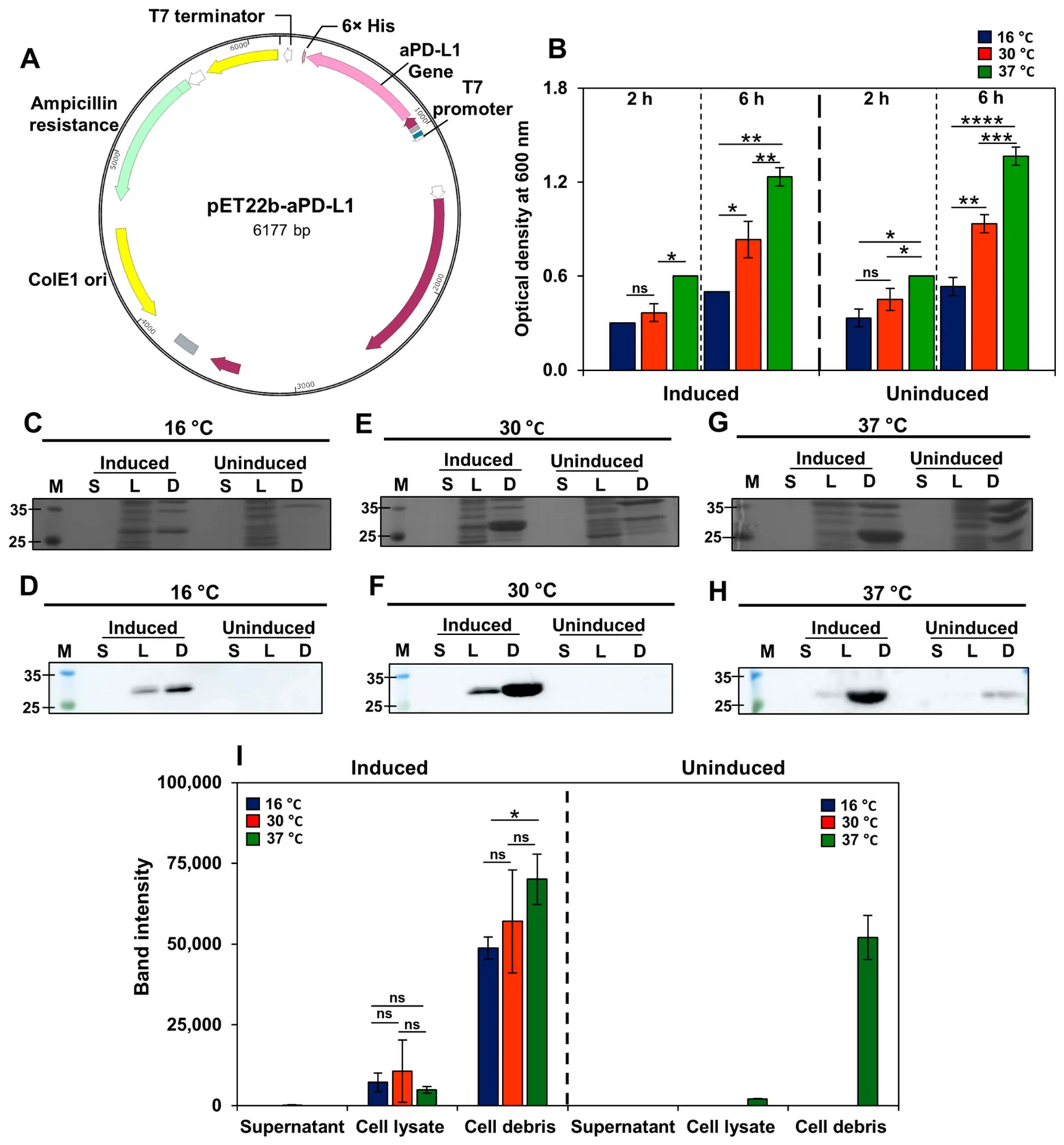
Expression of the aPD-L1 scFv in *E. coli* BL21(DE3). (**A**) A plasmid map used for aPD-L1 scFv expression in *E. coli* BL21(DE3). (**B**) Growth of *E. coli* BL21(DE3) at 2 h after inoculation (prior to induction) and 6 h after inoculation (4 h after induction) at different temperatures. The total proteins extracted from *E. coli* BL21(DE3) harboring pET-22b-aPD-L1 was used for (**C**,**E**,**G**) SDS-PAGE and (**D**,**F**,**H**) Western blot analysis in comparison between induced and uninduced cultures with 0.5 mM IPTG at 16 °C, 30 °C, and 37 °C, respectively. A total of 5 µg of protein was loaded into each well, except for the supernatant samples, where the total protein concentration was low; S = culture supernatant, L = soluble cell lysate and D = insoluble cell debris. (**I**) Densitometric analysis of the Western blot analysis using ImageJ software. Significant differences are indicated by asterisks (* *p* ≤ 0.05, ** *p* ≤ 0.01, *** *p* ≤ 0.001 and **** *p* ≤ 0.0001), while ns indicates no significant difference.

In this work, pET-22b(+) was used as an expression vector encoding the aPD-L1 scFv gene fused with C-terminal 6× histidine-tag (His-tagged aPD-L1 scFv) (Figure 1A). The plasmid was transformed into *E. coli* BL21(DE3) and, after 4 h of induction using 0.5 mM IPTG at different temperatures (16 °C, 30 °C, and 37 °C), no significant difference in growth was observed between cells with and without induction at each temperature. However, significant difference in growth was observed between temperatures by which higher growth was observed when higher temperature was used (Figure 1B). To investigate the solubility and localization of aPD-L1 scFv, cells were lysed by sonication, and the lysates were separated by centrifugation at 10,304× *g* at 4 °C for 30 min, yielding a soluble cell lysate (supernatant) and insoluble cell debris (pellet). Analyses by SDS-PAGE and Western blot showed that aPD-L1 scFv was expressed exclusively in induced cultures and was not detected in the culture supernatant at 16 °C and 30 °C, indicating that there was no leaky expression and aPD-L1 scFv remained inside of the cell (Figure 1C–F, Supplementary Figures S1–S4). However, leaky expression was observed at 37 °C (Figure 1G,H, Supplementary Figures S5 and S6). Densitometric analysis of the Western blot analysis suggested that, under these cultivation conditions, only 13%, 16% and 7% of the expressed protein was present in the soluble cell lysate, while the majority (87%, 84% and 93%) remained in the insoluble cell debris at 16 °C, 30 °C, and 37 °C, respectively (Figure 1I). Accordingly, 30 °C was chosen for further investigation.

### 3.2. Co-Expression of Molecular Chaperones with aPD-L1 scFv

**Figure 2:**
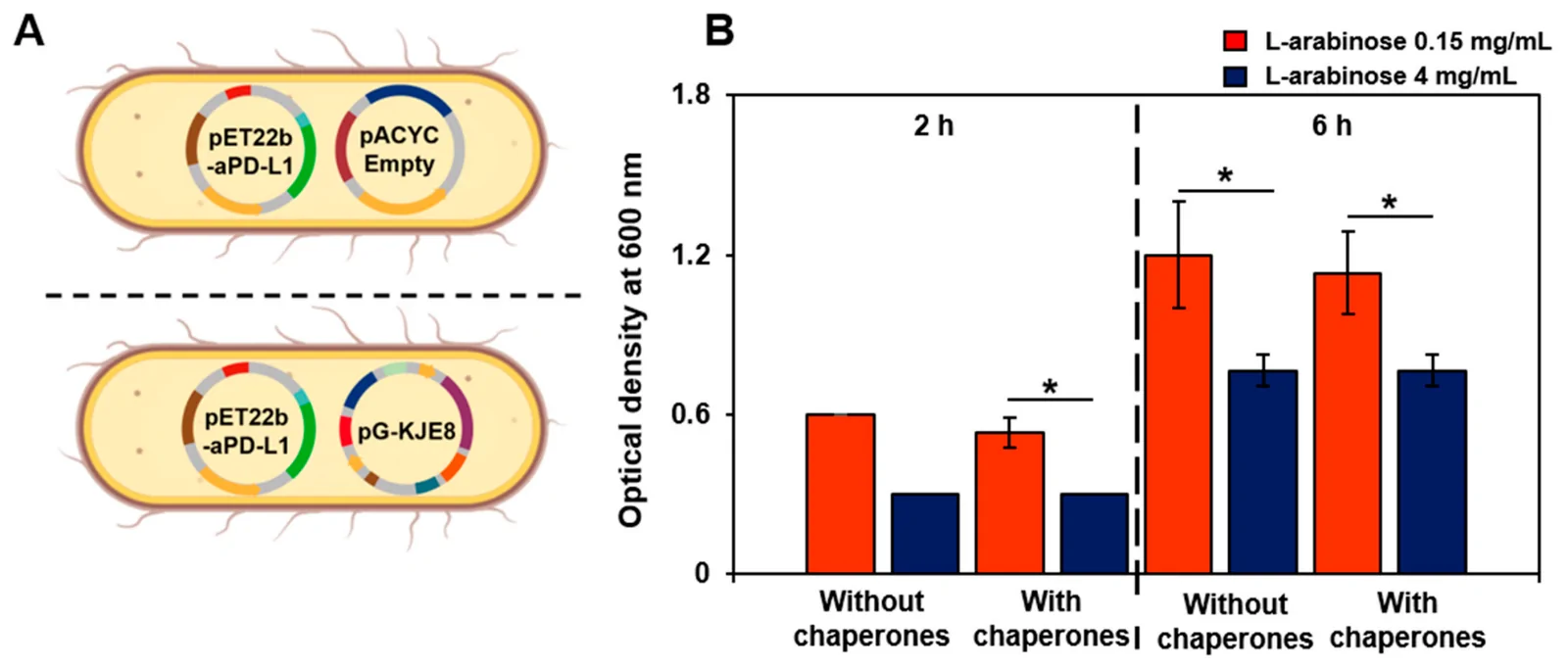
*E. coli* BL21(DE3) strains harboring different plasmids and their growth after 4 h of induction. (**A**) *E. coli* strains co-expressing aPD-L1 scFv with molecular chaperones and the control strain. (**B**) Growth of different strains at 2 h after inoculation (prior to induction) and 6 h after inoculation (4 h after induction) at different concentrations of L-arabinose at 0.15 mg/mL and 4 mg/mL. Significant differences are indicated by asterisks (*p* ≤ 0.05).

The pG-KJE8 plasmid expressing the molecular chaperones DnaK-DnaJ-GrpE and GroES-GroEL was co-transformed into *E. coli* BL21(DE3) harboring pET-22b-aPD-L1. An empty plasmid, pACYC, was co-transformed into the same strain as a control (Figure 2A). Different concentrations of L-arabinose as an inducer for DnaK-DnaJ-GrpE at 0.15 and 4 mg/mL were used as L-arabinose has been reported to alter *E. coli* cell growth [20] and the DnaK-DnaJ-GrpE acts at the early stage of protein folding by interacting with nascent polypeptide chains immediately after translation [21]. In contrast, tetracycline for the induction of GroES-GroEL was used at a fixed concentration of 5 ng/mL. Firstly, a significant difference in growth was observed, with higher cell growth in cultures induced with 0.15 mg/mL L-arabinose (Figure 2B).

**Figure 3:**
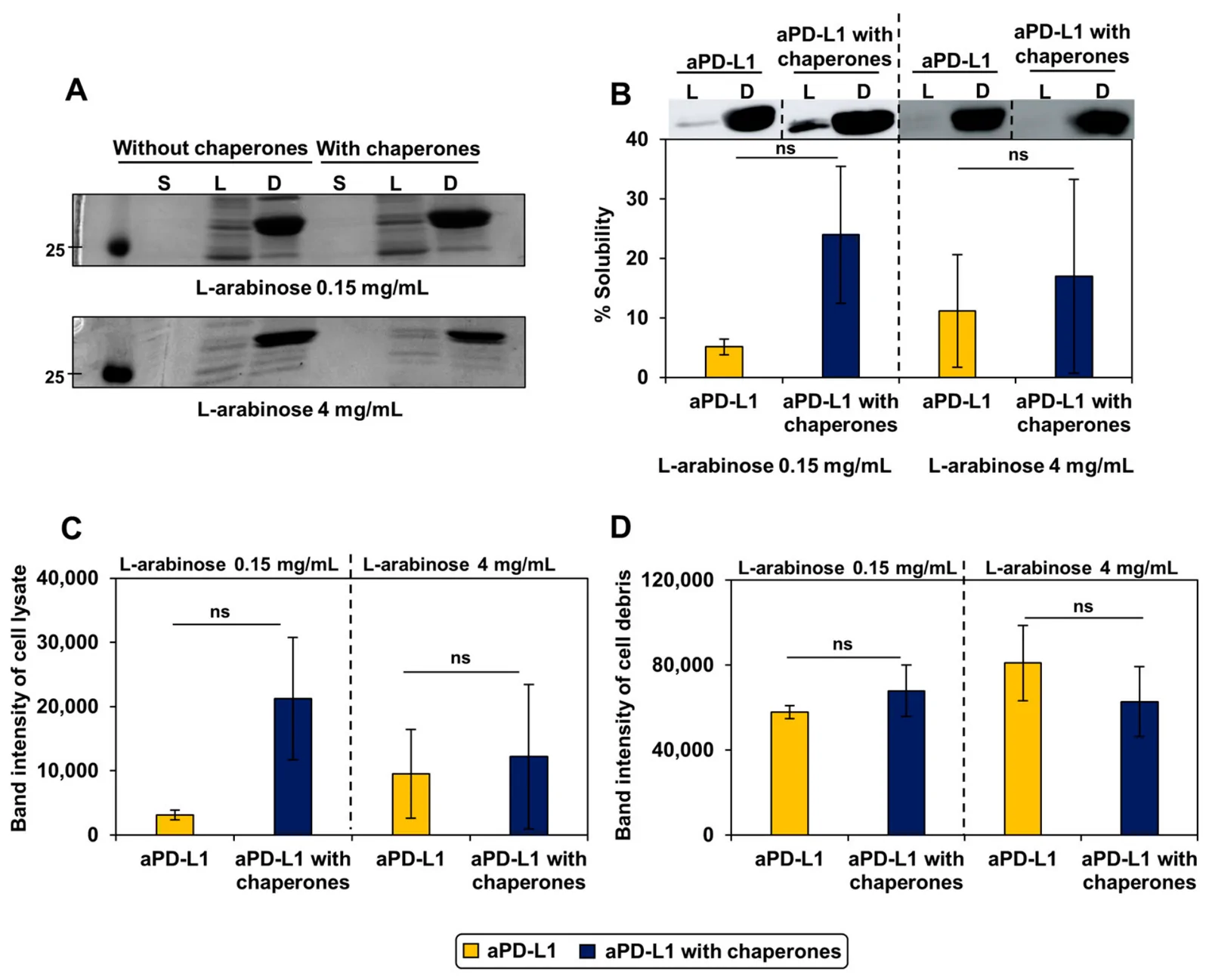
Co-expression of aPD-L1 with molecular chaperones. (**A**) SDS-PAGE analysis of total proteins extracted from *E. coli* BL21(DE3) co-expressing aPD-L1 without and with molecular chaperones, S = culture supernatant, L = soluble cell lysate and D = insoluble cell debris. (**B**) Densitometric analysis of the Western blot analysis from (**C**) soluble cell lysate and (**D**) insoluble cell debris fractions. ns indicates no significant difference.

Interestingly, when total proteins were extracted and analyzed using SDS-PAGE, the protein band at ~27 kDa was noticeably higher at 0.15 mg/mL of L-arabinose compared to 4 mg/mL. However, it was also observed that the majority amount of the proteins was localized in the insoluble cell debris fractions (Figure 3A and Supplementary Figure S7). Moreover, co-expression of aPD-L1 scFv with molecular chaperones resulted in higher solubility (23.97%) compared to expression without molecular chaperones (5.13%) when induced with 0.15 mg/mL L-arabinose, although the difference was not statistically significant. At a higher L-arabinose concentration (4 mg/mL), the increase was not as substantial, with solubility values of 11.20% and 16.99% observed for expression without and with molecular chaperones, respectively (Figure 3B and Supplementary Figure S8). These results were determined by densitometric analysis of Western blot analysis (Figure 3C,D).

### 3.3. Analysis of Purified aPD-L1 scFv

**Figure 4:**
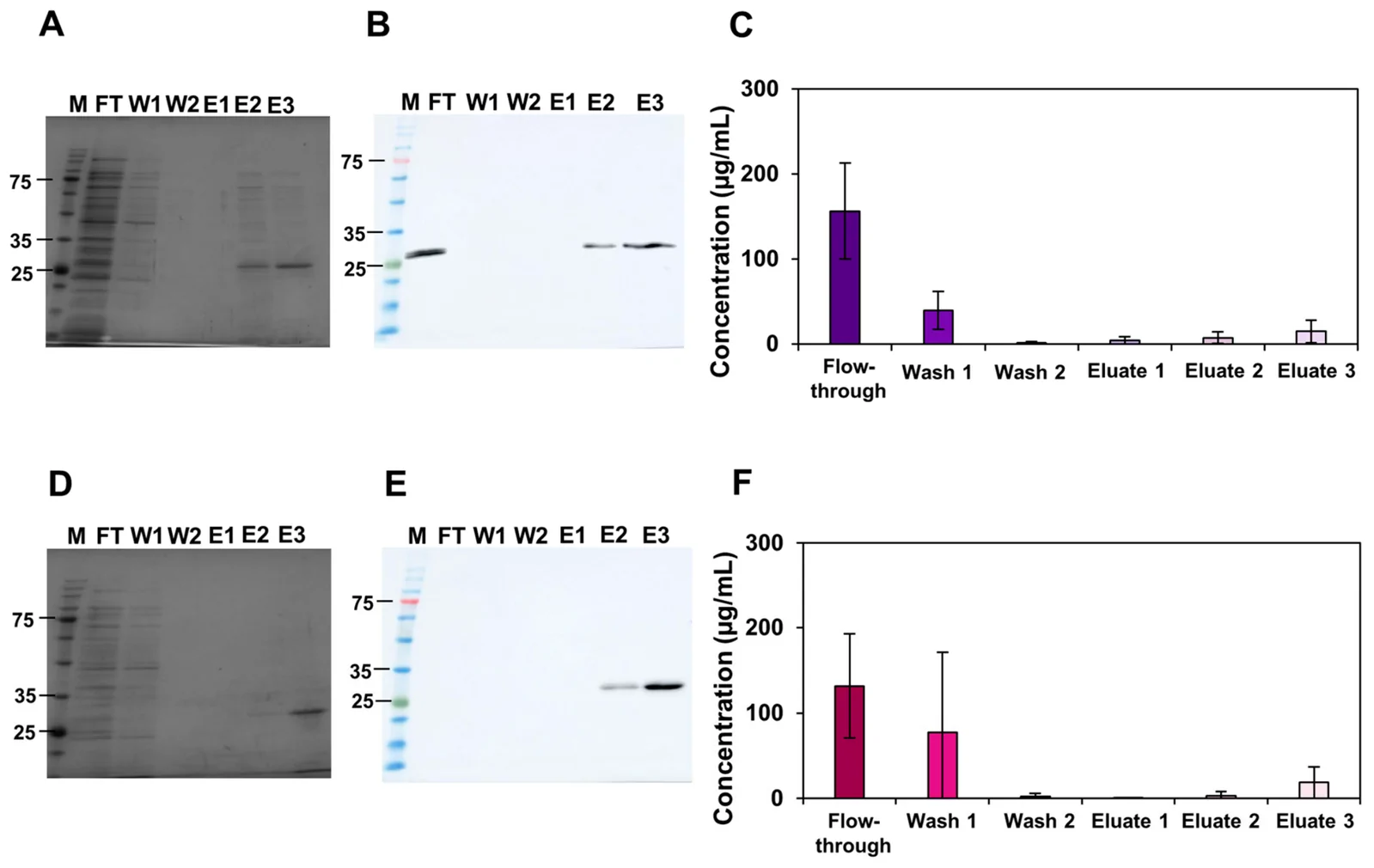
Protein purification using His GraviTrap TALON his-tag chromatography. SDS-PAGE and Western blot analysis of proteins extracted from (**A**,**B**) *E. coli* BL21(DE3) harboring pET-22b-aPD-L1 and pACYC and (**D**,**E**) *E. coli* BL21(DE3) harboring pET-22b-aPD-L1 and pG-KJE8. The concentrations of proteins from each fraction of purification from (**C**) *E. coli* BL21(DE3) harboring pET-22b-aPD-L1 and pACYC and (**F**) *E. coli* BL21(DE3) harboring pET-22b-aPD-L1 and pG-KJE8. The abbreviations indicate M = Marker, FT = Flow-through, W = Wash, E = Eluate.

The His-tagged aPD-L1 scFv was purified from the soluble cell lysate using His GraviTrap TALON His-tag affinity chromatography. SDS-PAGE analysis of all fractions (flow-through, wash, and elution) showed that the target protein (~27 kDa) was most abundant in the Eluate 3 fraction, which corresponded to the highest imidazole concentration (100 mM, 200 mM, and 400 mM for Eluate 1, Eluate 2, and Eluate 3, respectively), suggesting efficient elution at higher imidazole concentrations. Although a distinct band at the expected molecular weight of the target protein showed the highest intensity, additional protein bands of varying sizes were also observed in Eluate 3, indicating co-elution of non-specific proteins (Figure 4A,D and Supplementary Figure S9). Western blot analysis further confirmed that the purified His-tagged aPD-L1 scFv was primarily present in Eluate 3, with a weaker signal detected in Eluate 2 (Figure 4B,E and Supplementary Figure S10). Moreover, when the proteins were quantified, the protein concentration of the purified fraction was slightly higher in the treatment with the co-expression of molecular chaperones at 18.62 µg/mL compared with 14.78 µg/mL without the co-expression of molecular chaperones (Figure 4C,F); however, this was not significant. After desalting, the proteins were concentrated further to 56.73 and 85.27 µg/mL of the purified proteins without and with the co-expression of molecular chaperones, respectively.

### 3.4. Effect of Molecular Chaperone Co-Expression on aPD-L1 scFv Binding Activity

**Figure 5:**
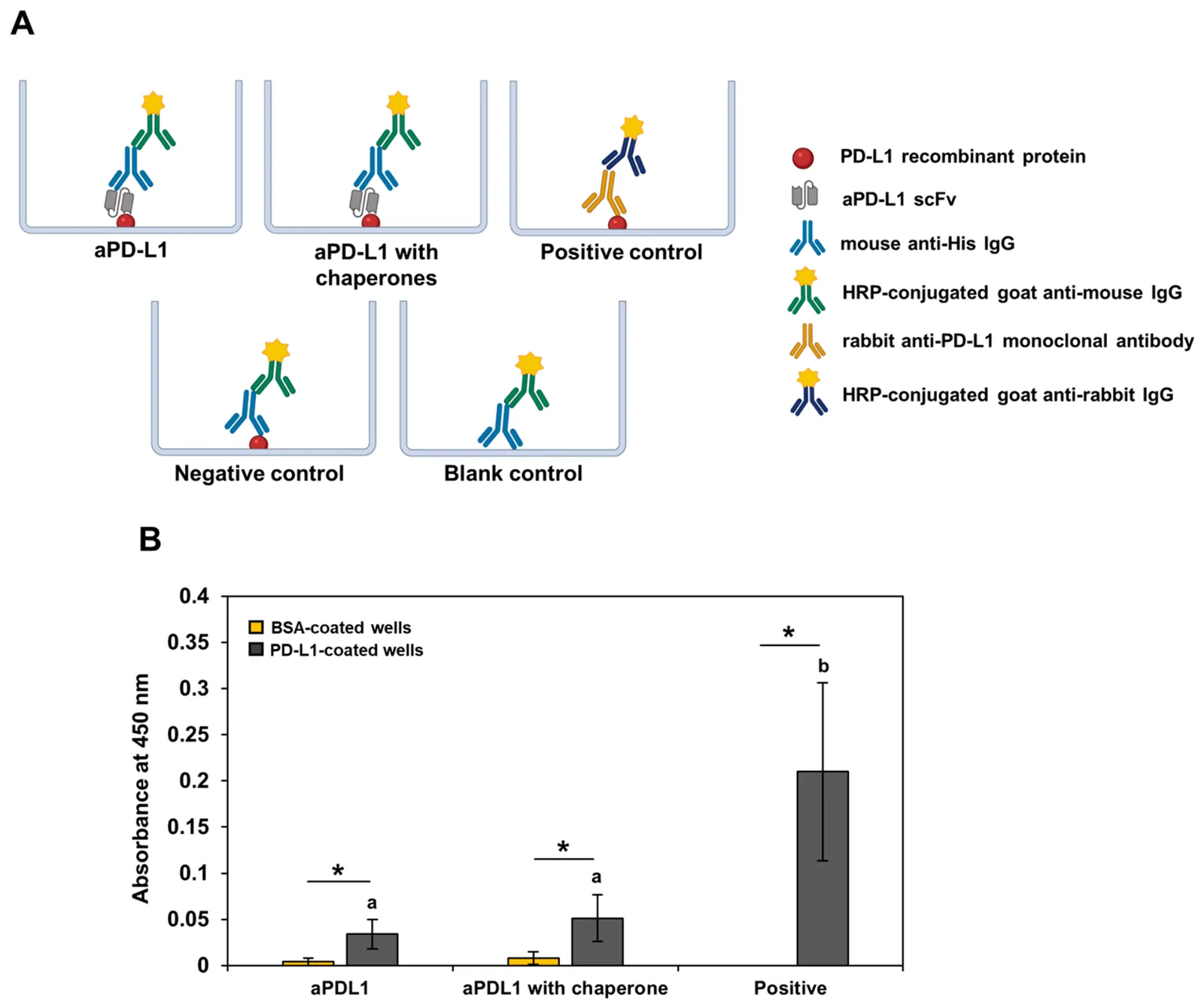
Effect of molecular chaperone co-expression on aPD-L1 scFv binding activity. (**A**) A schematic illustrating the comparative binding activity of aPD-L1 scFv via ELISA. Bovine serum albumin (BSA) was also included as an unrelated antigen. (**B**) OD450 after subtraction of negative control. Significant differences are indicated by asterisks (*p* ≤ 0.05). Different letters (a and b) indicate significant differences among the three groups in PD-L1-coated wells.

ELISA was performed to evaluate whether molecular chaperone co-expression affected the binding activity of purified aPD-L1 scFv, as molecular chaperones assist protein folding and may affect the structural conformation of the antibody variable regions responsible for antigen recognition. As illustrated in Figure 5A, purified aPD-L1 scFv without and with molecular chaperone co-expression were compared. A rabbit aPD-L1 monoclonal antibody (mAb), negative control and blank control were also included to validate the assay. The results showed that purified aPD-L1 scFv exhibited comparable binding activity regardless of molecular chaperone co-expression, with no significant difference observed between the two conditions (Figure 5B). Both aPD-L1 scFv preparations exhibited higher binding signals toward PD-L1-coated wells than toward BSA-coated wells, supporting preferential recognition of the target antigen. A commercially available rabbit aPD-L1 monoclonal antibody was included as a positive control to validate the ELISA by confirming binding to recombinant human PD-L1. However, it was not intended as a direct comparator to the recombinant aPD-L1 scFv. The higher ELISA signal observed for the positive control is likely attributable to differences in antibody format and sequence, as the positive control is a full-length IgG whereas the recombinant antibody produced in this study is an scFv.

## 4. Discussion

Although a few studies have reported successful expression of biologically functional aPD-L1 scFv in *E. coli* [22,23,24], the solubility of the expressed scFv has not been systematically quantified. Specifically, previous reports described yields based only on the recovered soluble fractions, without comparison to total expression levels. In our study, it was shown that *E. coli* BL21(DE3) produced only 16% soluble form of the aPD-L1 scFv indicating that 84% of the aPD-L1 scFv formed inclusion bodies. Therefore, establishing a robust production strategy to increase soluble expression would lead to the broader applicability of this platform for other antibody fragments. Interestingly, the comparable expression levels observed in induced and uninduced cultures at 37 °C may be attributed to basal (leaky) expression of the T7 promoter, a known feature of T7-based expression systems in *E. coli*. Basal expression of T7 RNA polymerase from the lacUV5 promoter can drive transcription of the target gene from the T7 promoter even in the absence of IPTG induction [25]. This pattern was not observed at 16 °C and 30 °C, suggesting that cultivation temperature may influence the extent to which basal expression becomes detectable, although the underlying mechanism was not investigated in this study.

To improve the soluble expression of aPD-L1 scFv, molecular chaperones DnaK-DnaJ-GrpE and GroES-GroEL were co-expressed with aPD-L1 scFv. The results suggested that at the lower L-arabinose concentration (0.15 mg/mL), the % solubility increased from 5.13% to 23.97% when co-expressed with molecular chaperones, although the difference was not statistically significant. A previous study has reported the use of DnaK-DnaJ-GrpE and GroES-GroEL in the same combination for the expression of anti-EGFR huscFv. The % solubility was reported to increase from 26% (without co-expression) to 54% (with co-expression) [26]. Moreover, in the present study, it was shown that significantly lower growth was observed when 4 mg/mL of L-arabinose was added to the cultures. It has previously been reported that L-arabinose could alter the growth behavior of *E. coli* by increasing the planktonic growth rate while reducing total biomass accumulation [20]. Different L-arabinose concentrations have been employed to induce molecular chaperone expression from the pG-KJE8 plasmid, depending on the target protein and expression conditions. For instance, 0.5 mg/mL L-arabinose was used to induce DnaK-DnaJ-GrpE expression for recombinant papain production and showed improved folding efficiency and soluble protein yield [27]. In contrast, a lower concentration of 0.09 mg/mL was applied for co-expression with anti-EGFR huscFv [26]. Additionally, a concentration of 0.15 mg/mL L-arabinose has previously been used for chaperone induction in *E. coli,* resulting in enhanced soluble and functional expression of anti-BNP scFv [12]. These findings suggest that molecular chaperone expression from pG-KJE8 is highly tunable and does not require high L-arabinose concentrations to be effective. Instead, lower inducer concentrations may be sufficient and could help reduce metabolic burden while still enhancing protein folding efficiency.

Importantly, along with the % solubility, which is a key to molecular chaperone co-expression, the amount of purified aPD-L1 scFv is another indicator. The results indicated that co-expression with molecular chaperones slightly increased the protein concentration of purified aPD-L1 scFv, increasing the concentrations from 14.78 µg/mL to 18.62 µg/mL. The slight increase in purified aPD-L1 scFv resulting from molecular chaperone co-expression is expected, as molecular chaperones can enhance the proportion of properly folded recombinant proteins in the soluble cell lysate by reducing aggregation and inclusion body formation. Since the purified fraction was obtained from the soluble lysate, improved protein solubility could increase the amount of recombinant aPD-L1 scFv available for downstream affinity purification and may therefore contribute to the observed difference in purified protein concentration. However, variation in protein recovery during the purification process may have contributed to the variability in the final protein concentration, resulting in a difference that did not reach statistical significance. Interestingly, these concentrations are considered relatively high compared with a previous work that reported the purified aPD-L1 scFv at 5.64 µg/mL from *E. coli* SHuffle^®^ strain [23]. However, a recent study reported a purified aPD-L1 scFv-Fc yield of approximately 80–100 μg/L using *E. coli* Rosetta(DE3) and *E. coli* SHuffle^®^ under similar expression conditions [24]. Direct comparison should be made with caution because the construct design and expression systems were different. In the previous study, an aPD-L1 scFv-Fc fusion construct was expressed using the pET-32a vector in *E. coli* Rosetta(DE3) and *E. coli* SHuffle^®^, whereas the present study used a pET-22b(+) vector to express aPD-L1 scFv in *E. coli* BL21(DE3) together with molecular chaperone co-expression. These differences in the expression construct, vector backbone, and host strain are likely to influence protein folding, solubility, and ultimately the recombinant protein yield. Nevertheless, the previous study suggests that further optimization, particularly by exploring alternative *E. coli* strains and expression strategies, may further improve aPD-L1 scFv production [24]. Moreover, future studies should further improve reproducibility by tightly controlling expression conditions and normalizing the amount of total proteins prior to affinity purification to minimize variation between biological replicates.

It is important to validate whether the chaperone-co-expressed aPD-L1 scFv could maintain the binding activity to strengthen its further use. The results showed that the binding activity of the aPD-L1 scFv at 20 µg/mL was similar with and without co-expression. This observation is consistent with the primary role of molecular chaperones in increasing the yield of soluble protein by preventing aggregation and facilitating proper folding, rather than altering the intrinsic binding properties of correctly folded scFv molecules. Therefore, the comparable ELISA signals indicate that chaperone co-expression improved soluble protein production while preserving the functional activity of the purified aPD-L1 scFv. Moreover, although formation of the conserved intradomain disulfide bonds in the VH and VL domains may be unfavorable in the reducing cytoplasm of *E. coli* BL21(DE3) [28], the retained binding activity suggests that the scFv can adopt a functional conformation under these conditions. However, the disulfide bond status of the expressed aPD-L1 scFv was not directly determined in this study. In addition, the binding activity of the purified aPD-L1 scFv was evaluated at a single concentration (20 µg/mL); therefore, concentration-dependent binding and EC50 values were not determined. Further studies using a range of scFv concentrations would enable more quantitative characterization of its binding activity. It should also be noted that the commercial rabbit aPD-L1 monoclonal antibody was included as a positive control to validate the ELISA. Its higher signal compared with the purified aPD-L1 scFv is likely attributable to differences in antibody format, as the positive control is a full-length IgG whereas the antibody produced in this study is an scFv with different structural and binding properties. Although no studies have reported the use of molecular chaperones for the expression of aPD-L1 scFv, a previous study comparing different combinations of molecular chaperones demonstrated that co-expression of ABA-scFv with DnaK-DnaJ-GrpE and GroES-GroEL did not result in a significant difference in binding activity relative to the control without the co-expression, as assessed by ELISA [29]. In contrast, a comparison of different sets of molecular chaperones for the expression of anti-BNP scFv showed that co-expression with DnaK-DnaJ-GrpE and GroES-GroEL resulted in lower binding activity, whereas expression with GroES-GroEL alone or trigger factor (TF) enhanced binding activity [12]. This may suggest that the effect of molecular chaperone co-expression on scFv functionality is highly dependent on the specific antibody and folding environment, and that optimal molecular chaperone combinations may vary accordingly. Moreover, the aPD-L1 scFv used in this study was derived from a previously published aPD-L1 scFv sequence originally developed for a BiTE construct [19]. Although its precise epitope has not been mapped, the original study demonstrated that this scFv competitively inhibited the binding of a commercial aPD-L1 monoclonal antibody to PD-L1, suggesting that it recognizes overlapping or closely located binding sites. Nevertheless, detailed epitope mapping is required to confirm the exact binding site.

To further enhance soluble scFv production, multiple engineering strategies can be combined, each targeting different bottlenecks in recombinant protein expression. Molecular chaperone co-expression primarily facilitates protein folding by preventing aggregation and promoting the acquisition of native conformations. As described above, the DnaK-DnaJ-GrpE system used in this work assists nascent polypeptides through ATP-dependent binding and release cycles, whereas GroES-GroEL provides an isolated folding chamber for newly synthesized or misfolded proteins. In contrast, host strain engineering improves expression through distinct mechanisms. A limitation of this study is that the molecular chaperone co-expression experiments were conducted only in *E. coli* BL21(DE3). The *E. coli* SHuffle^®^ strain promotes the correct folding of disulfide-rich proteins by providing an oxidizing cytoplasmic environment and expressing the disulfide bond isomerase DsbC, making it particularly suitable for antibody fragments such as scFvs [30]. Conversely, the *E. coli* Rosetta(DE3) strain enhances translation efficiency by supplying tRNAs for codons that are rare in *E. coli*, rather than directly improving protein folding. A previous comparison of expression systems for aPD-L1 scFv demonstrated that *E. coli* SHuffle^®^ produced higher soluble yields than *E. coli* Rosetta(DE3) [24], while another study similarly reported improved soluble expression of anti-EpCAM scFv in *E. coli* SHuffle^®^ compared with *E. coli* BL21(DE3) [31]. These observations suggest that host strain engineering and molecular chaperone co-expression represent complementary approaches that address different stages of recombinant protein production. Future studies will therefore compare other *E. coli* expression strains, such as SHuffle^®^ and Rosetta(DE3), to evaluate strain-dependent effects on aPD-L1 scFv production.

## 5. Conclusions

Here, *E. coli* was developed as a production platform for aPD-L1 scFv as an alternative to mammalian platforms. *E. coli* BL21(DE3) was engineered to harbor pET-22b-aPD-L1 for the expression of aPD-L1 scFv. The results showed that the expression of aPD-L1 scFv alone exhibited a high % insoluble expression. Molecular chaperone co-expression resulted in a numerically higher aPD-L1 scFv solubility ratio (23.97%) compared with expression without chaperones (5.13%), although no statistically significant difference was observed. Moreover, the purification of aPD-L1 scFv showed that *E. coli* BL21(DE3) produced aPD-L1 scFv at 14.78 µg/mL compared with 18.62 µg/mL without and with co-expression with molecular chaperones, respectively. Binding activity was also investigated, and the results showed that the expressed aPD-L1 scFv retained its activity, suggesting that co-expression did not affect binding. In the future, to further improve the solubility of the aPD-L1 scFv, different strains of *E. coli* that have been demonstrated to produce more soluble recombinant proteins could be the next strategy.

## Figures and Tables

**Table 1:** A list of plasmids used in this study.

Plasmid/Purpose	Encoded Gene	Promoter/ Inducer	Origin of Replication	Selectable Marker	Source
pET-22b(+)/ expression vector backbone	-	T7/IPTG	ColE1	Amp^R^	Sigma-Aldrich (St. Louis, MO, USA)
pET-22b-aPD-L1/ aPD-L1 scFv expression plasmid	*aPD-L1* *scFv*	T7/IPTG	ColE1	Amp^R^	Constructed in this study
pACYC/ empty vector control	-	-	p15A	Cm^R^	GoldBio
pG-KJE8/ chaperone expression plasmid	*dnaK-dnaJ-grpE and groES-groEL*	araB/ L-Arabinose and Pzt-1/ Tetracycline	p15A	Cm^R^	Takara Bio

## Data Availability

All data generated or analyzed during this study are included in this published article and its Supplementary Information Files.

## Supplementary Materials

The following supporting information can be downloaded at: https://www.mdpi.com/article/10.3390/life16101664/s1, Figure S1: Full SDS-PAGE analysis of aPD-L1 scFv expression in *E. coli* BL21(DE3) following induction at 16 °C; Figure S2: Full Western blot analysis of aPD-L1 scFv expression in *E. coli* BL21(DE3) following induction at 16 °C; Figure S3: Full SDS-PAGE analysis of aPD-L1 scFv expression in *E. coli* BL21(DE3) following induction at 30 °C; Figure S4: Full Western blot analysis of aPD-L1 scFv expression in *E. coli* BL21(DE3) following induction at 30 °C; Figure S5: Full SDS-PAGE analysis of aPD-L1 scFv expression in *E. coli* BL21(DE3) following induction at 37 °C; Figure S6: Full Western blot analysis of aPD-L1 scFv expression in *E. coli* BL21(DE3) following induction at 37 °C; Figure S7: Full SDS-PAGE analysis of co-expression of aPD-L1 scFv with molecular chaperones; Figure S8: Full Western blot analysis of co-expression of aPD-L1 scFv with molecular chaperones; Figure S9: Full SDS-PAGE analysis of protein purification using His GraviTrap TALON; Figure S10: Full Western blot analysis of protein purification using His GraviTrap TALON.
