# Supplementary material for: Co-Expression of Molecular Chaperones to Enhance Soluble aPD-L1 scFv Production in Escherichia coli BL21(DE3)
Source: Life (Basel). 2026 Oct 3;16(10):1664. doi: 10.3390/life16101664 (PMC13705652; doi:10.3390/life16101664)
Supplement: Supplementary file 1 [file life-16-01664-s001.zip › life-4587052-supplementary.pdf]

Supplementary information

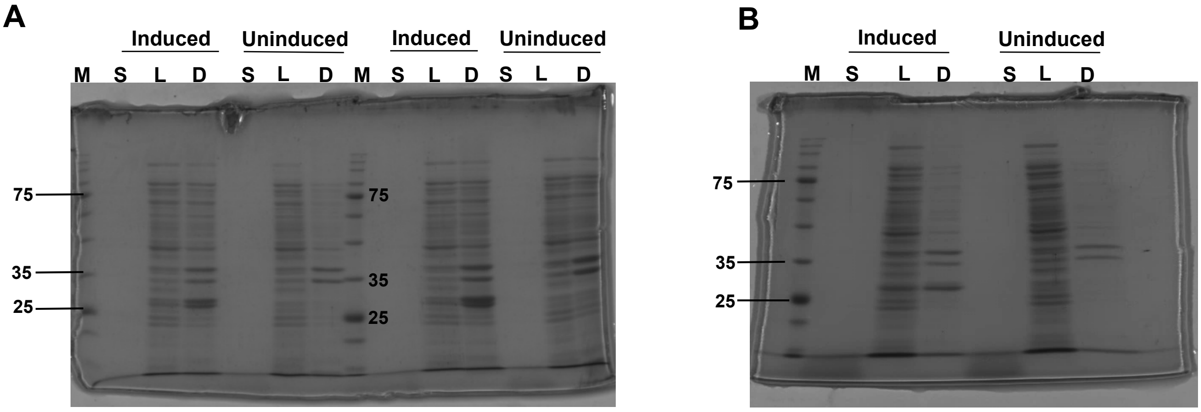

Figure S1 Full SDS-PAGE analysis of aPD-L1 scFv expression in *E. coli* BL21(DE3) following induction at 16°C. Panels A and B show the uncropped gels from three independent biological replicates. M: protein marker; S: supernatant; L: lysate; D: debris. Induced and uninduced samples are indicated above each lane.

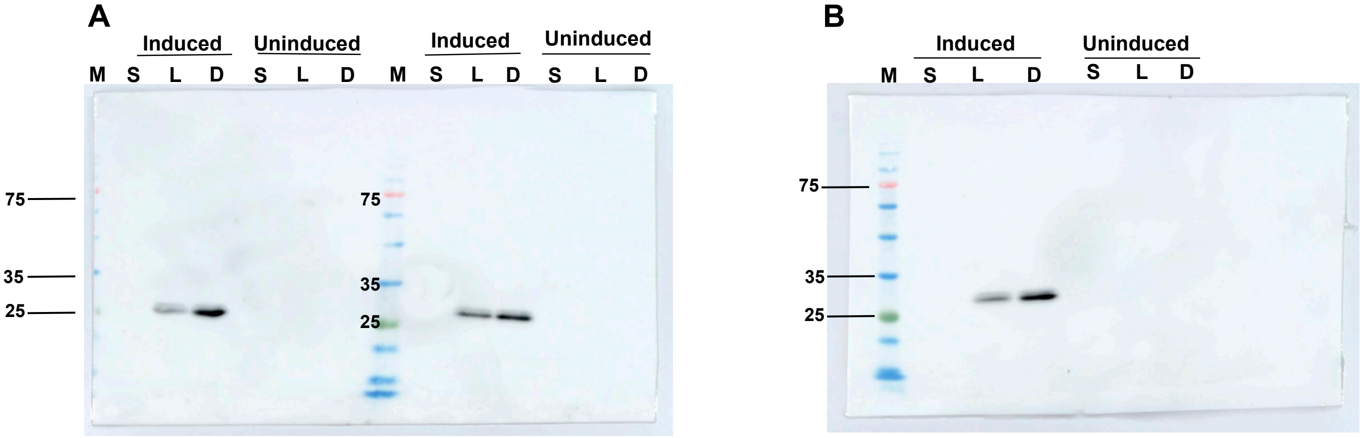

Figure S2 Full Western blot analysis of aPD-L1 scFv expression in *E. coli* BL21(DE3) following induction at 16°C. Panels A and B show the uncropped gels from three independent biological replicates. M: protein marker; S: supernatant; L: lysate; D: debris. Induced and uninduced samples are indicated above each lane.

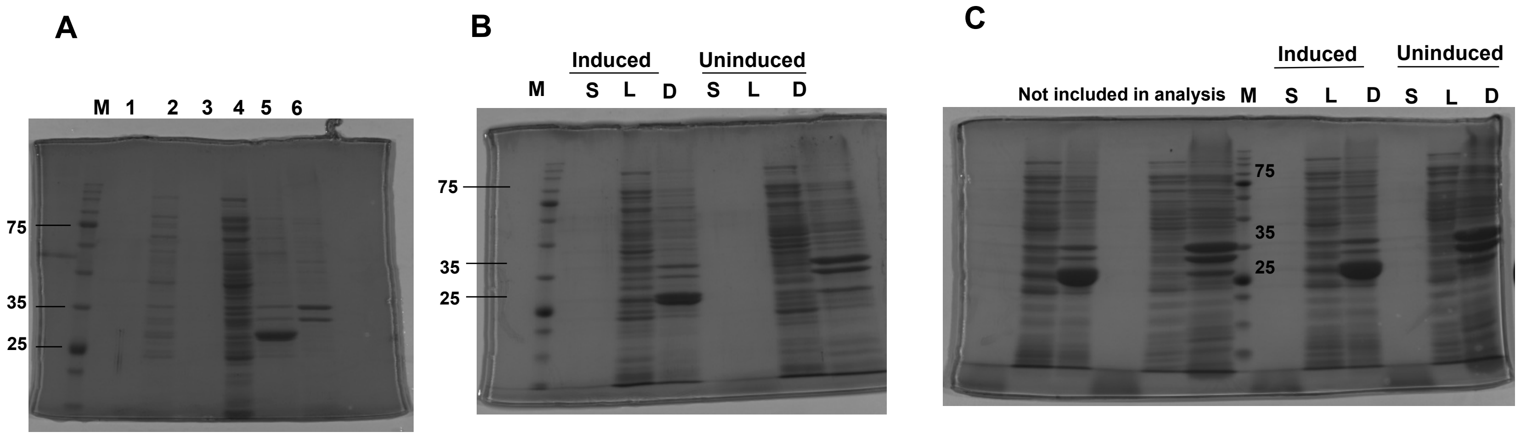

Figure S3 Full SDS-PAGE analysis of aPD-L1 scFv expression in *E. coli* BL21(DE3) following induction at 30°C. (A-C) show the uncropped gels from three independent biological replicates. **Panel A** Lane 1: supernatant induced, Lane 2: supernatant uninduced, Lane 3: lysate induced; Lane 4: lysate uninduced, Lane 5: debris induced, Lane 6: debris uninduced. **Panel B and C** show the uncropped gels from three independent biological replicates. M: protein marker; S: supernatant; L: lysate; D: debris. Induced and uninduced samples are indicated above each lane.

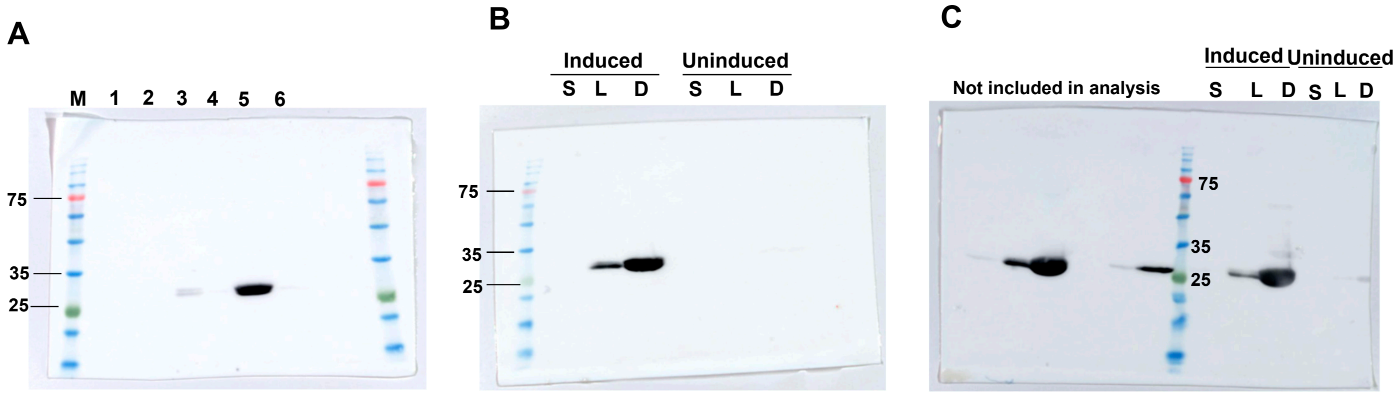

Figure S4 Full Western blot analysis of aPD-L1 scFv expression in *E. coli* BL21(DE3) following induction at 30°C. (A-C) show the uncropped gels from three independent biological replicates. **Panel A** Lane 1: supernatant induced, Lane 2: supernatant uninduced, Lane 3: lysate induced; Lane 4: lysate uninduced, Lane 5: debris induced, Lane 6: debris uninduced. **Panel B and C** show the uncropped gels from three independent biological replicates. M: protein marker; S: supernatant; L: lysate; D: debris. Induced and uninduced samples are indicated above each lane.

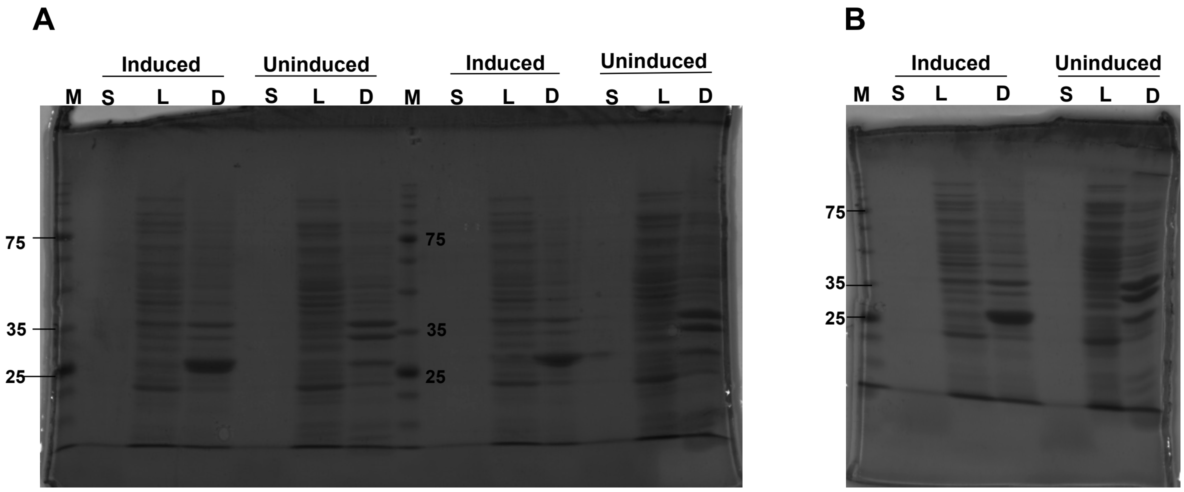

Figure S5 Full SDS-PAGE analysis of aPD-L1 scFv expression in *E. coli* BL21(DE3) following induction at 37°C. Panels A and B show the uncropped gels from three independent biological replicates. M: protein marker; S: supernatant; L: lysate; D: debris. Induced and uninduced samples are indicated above each lane.

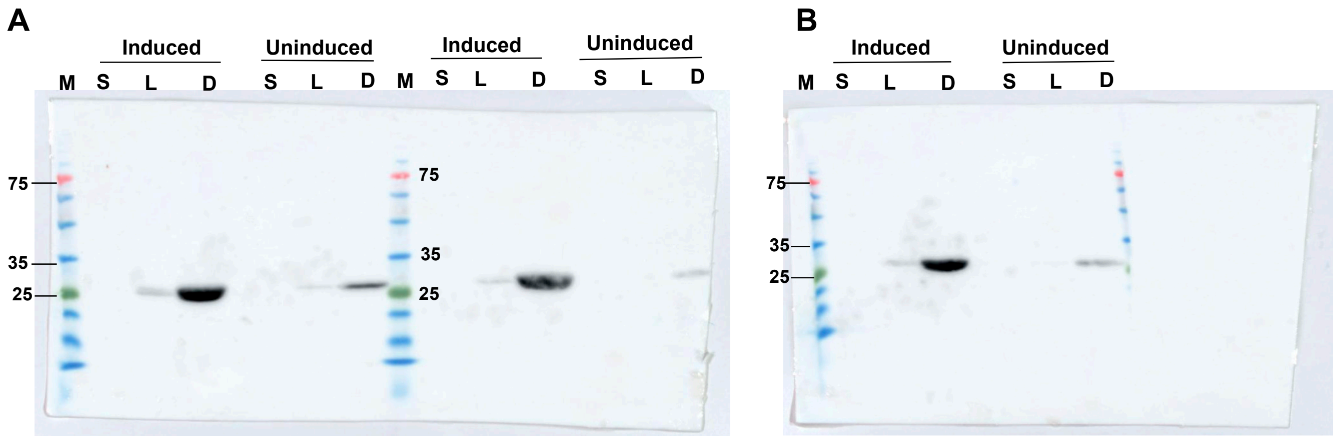

Figure S6 Full Western blot analysis of aPD-L1 scFv expression in *E. coli* BL21(DE3) following induction at 37°C. Panels A and B show the uncropped gels from three independent biological replicates. M: protein marker; S: supernatant; L: lysate; D: debris. Induced and uninduced samples are indicated above each lane.

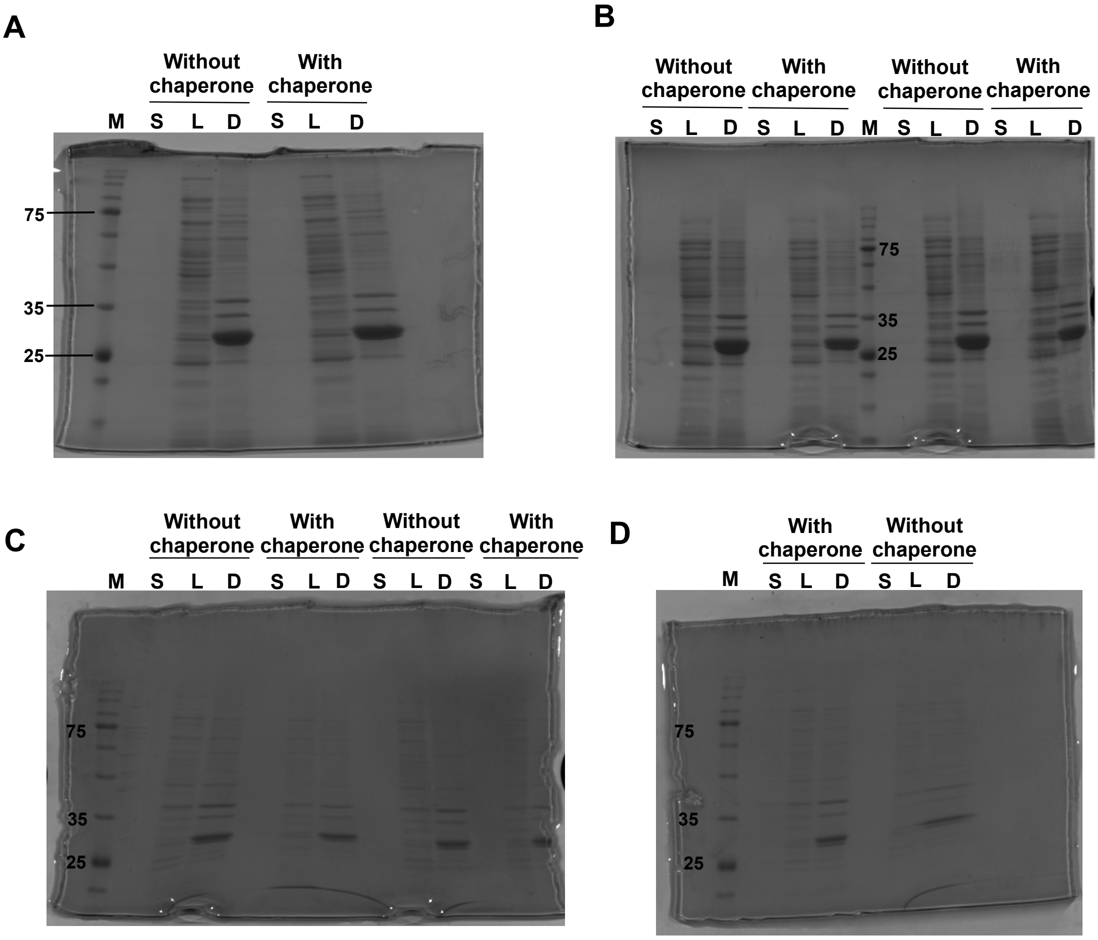

Figure S7 Full SDS-PAGE analysis of aPD-L1 scFv co-expression with molecular chaperones under different L-arabinose concentrations. (A–C) Representative full SDS-PAGE images of cells induced with 0.15 mg/mL L-arabinose. **(C–D)** Representative full Western blot images of cells induced with 4 mg/mL L-arabinose. Without chaperone indicates cells carrying pET-22b-aPD-L1 and pACYC, whereas with chaperone indicates cells carrying pET-22b-aPD-L1 and pG-KJE8. M, protein marker; S, supernatant; L, lysate; D, debris.

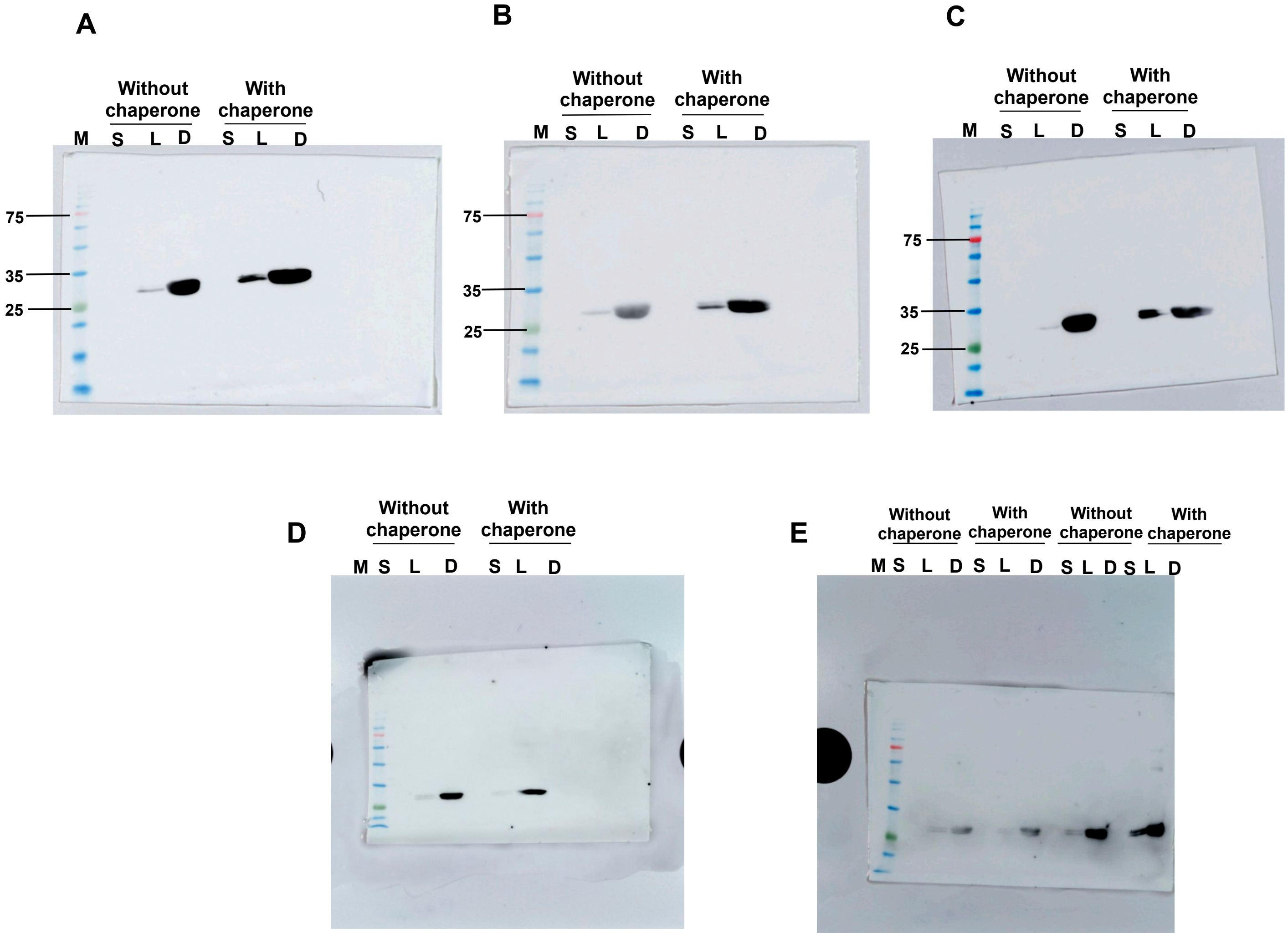

Figure S8 Full Western blot analysis of aPD-L1 scFv co-expression with molecular chaperones under different L-arabinose concentrations. (A–C) Representative full Western blot images of cells induced with 0.15 mg/mL L-arabinose. **(D–E)** Representative full Western blot images of cells induced with 4 mg/mL L-arabinose. Without chaperone indicates cells carrying pET-22b-aPD-L1 and pACYC, whereas with chaperone indicates cells carrying pET-22b-aPD-L1 and pG-KJE8. M, protein marker; S, supernatant; L, lysate; D, debris.

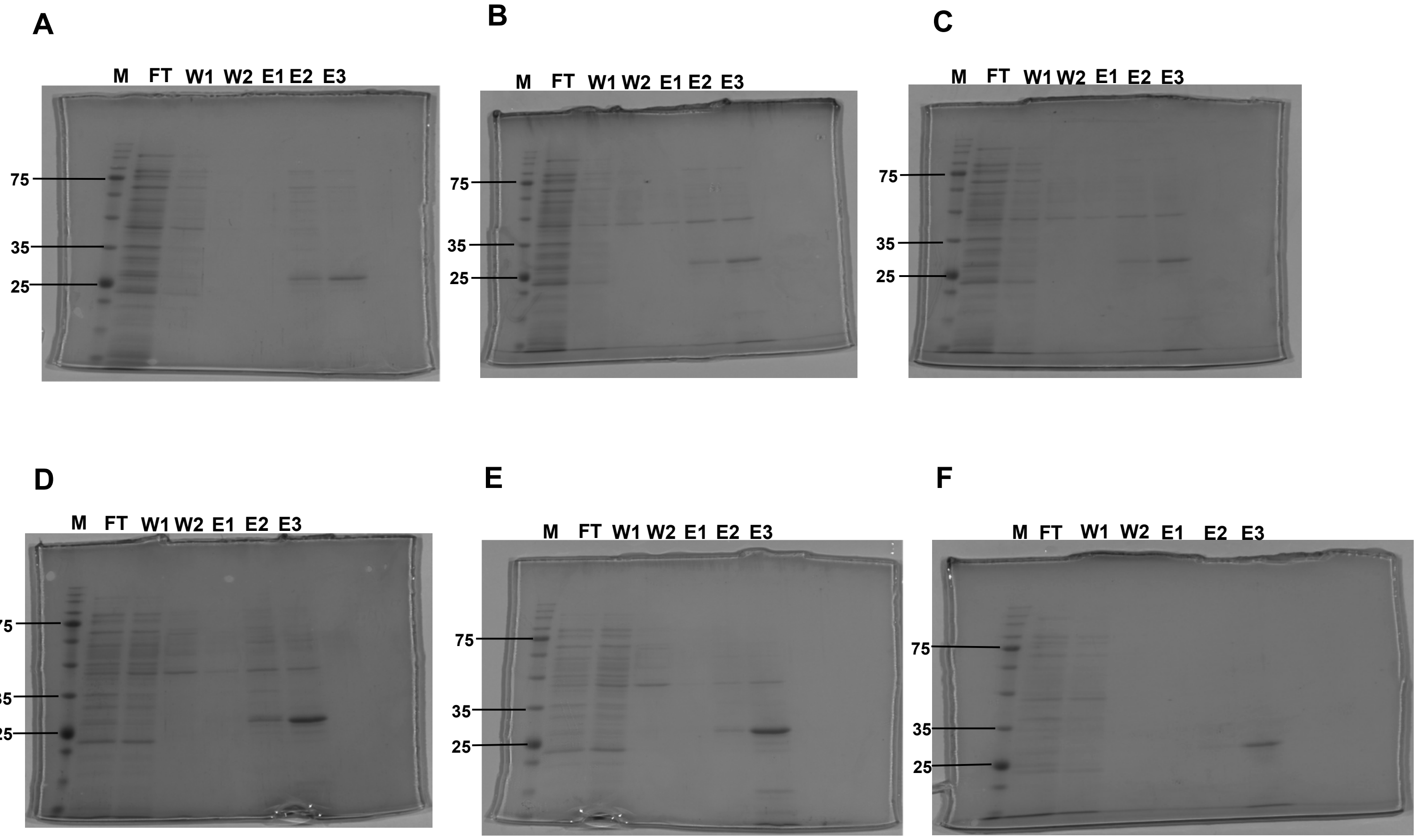

Figure S9 Full SDS-PAGE Protein purification using His GraviTrap TALON. (A–C) Purification of aPD-L1 scFv co-expressed without molecular chaperones. **(D–F)** Purification of aPD-L1 scFv co-expressed with molecular chaperones. M: Protein marker, FT: Flow-through, W1: Wash 1, W2: Wash 2, E1: Eluate 1, E2: Eluate 2, E3: Eluate 3.

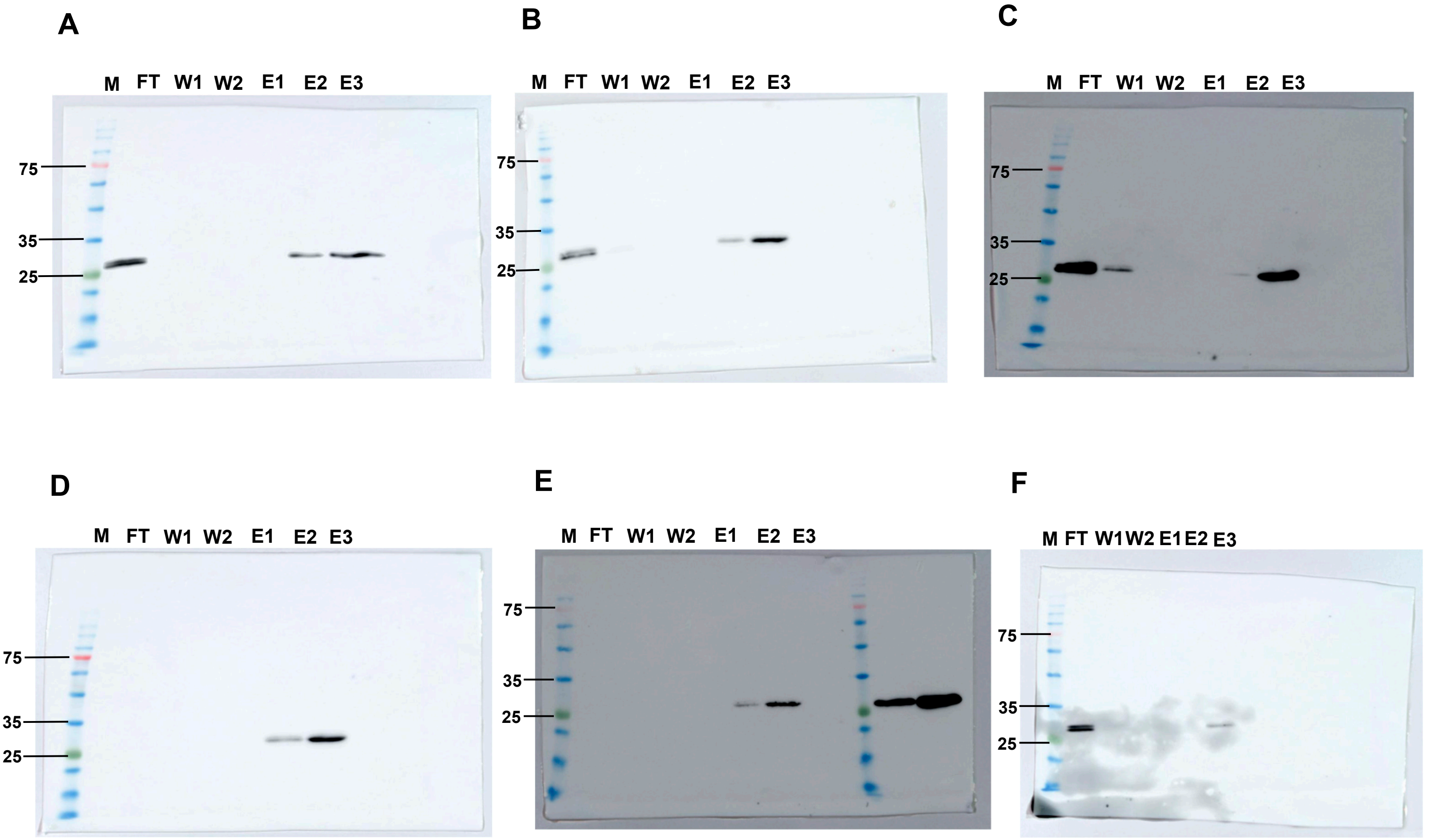

Figure S10 Full Western blot Protein purification using His GraviTrap TALON. (A–C) Purification of aPD-L1 scFv co-expressed without molecular chaperones. **(D–F)** Purification of aPD-L1 scFv co-expressed with molecular chaperones. M: Protein marker, FT: Flow-through, W1: Wash 1, W2: Wash 2, E1: Eluate 1, E2: Eluate 2, E3: Eluate 3.
